# Beyond symptom: Exploring the analgesic properties of vomiting in patients with migraine

**DOI:** 10.1111/head.70106

**Published:** 2026-05-27

**Authors:** Antonio Munafò, Andrea Burgalassi, Giulia Vigani, Luigi Francesco Iannone, Francesco De Cesaris, Alberto Chiarugi

**Affiliations:** ^1^ Headache Center and Clinical Pharmacology Unit Careggi University Hospital Florence Italy; ^2^ Section of Clinical Pharmacology and Oncology, Department of Health Sciences University of Florence Florence Italy; ^3^ Digital and Predictive Medicine, Pharmacology and Clinical Metabolic Toxicology‐Headache Center and Drug Abuse‐Laboratory of Clinical Pharmacology and Pharmacogenomics AOU Policlinico Di Modena Modena Italy; ^4^ Department of Biomedical, Metabolic, and Neural Science University of Modena and Reggio Emilia Modena Italy

**Keywords:** endogenous analgesia, migraine, self‐medication behavior, vomiting‐induced pain relief

## Abstract

**Objective:**

This study was conducted to characterize the prevalence, temporal patterns, and the degree of vomiting‐induced migraine pain relief (VIMPR).

**Background:**

Although data in the literature and anecdotal patient reports indicate the occurrence of VIMPR, its detailed characterization remains unstudied. Knowledge gaps are present regarding the extent and the temporal dynamics of pain relief upon vomiting in patients with migraine.

**Methods:**

We conducted an explorative, cross‐sectional, questionnaire‐based study on patients with migraine referring at the Headache Center of Careggi University Hospital, Florence, Italy (June–December 2023). We assessed vomiting frequency, number of episodes, pain intensity (numerical rating scale [0–10]) for attacks with and without vomiting, temporal relationship between vomiting and VIMPR, pain recurrence patterns, as well as vomiting induction behaviors.

**Results:**

Among 106 patients with migraine, 82 of 106 (77.4%; 95% confidence interval [CI], 68.5%–84.3%) reported vomiting during attacks. Attacks with vomiting were significantly more painful than those without (median, 9.0 vs. 7.0, Wilcoxon signed‐rank test, *V* = 70, *p* < 0.001, *r* = 0.77). VIMPR occurred in 45 of 82 patients (54.9%; 95% CI, 44.1%–65.2%), with 12 of 82 (14.6%) experiencing complete attack cessation whereas 33 of 82 (40.2%) only temporary reduction. Of the 45 patients experiencing VIMPR, onset of pain relief occurred within seconds in 13 of 45 (28.9%), within minutes in 18 of 45 (40.0%), and within hours in 14 of 45 (31.1%). For those with pain recurrence, 57.6% experienced reduced intensity compared to pre‐vomiting levels. Voluntary vomiting induction was reported by 22 of 82 patients (26.8%; 95% CI, 18.4%–37.3%) and was more common among those who had experienced VIMPR (18 of 45, 40.0%) compared to those without VIMPR (4 of 37, 10.8%; χ^2^(1) = 7.39, *p* = 0.007).

**Conclusions:**

Vomiting seems to provide pain relief and/or pain freedom in more than half of patients with migraine. Although the cross‐sectional, self‐reported nature of our data does not allow definitive conclusions regarding underlying mechanisms, the rapid onset of VIMPR reported by patients and the learned behavior of voluntary vomiting induction are consistent with the involvement of endogenous antinociceptive pathways. Future studies employing neuroimaging techniques, prospective designs, along with the consistent documentation of acute medication use, are needed to elucidate whether VIMPR represents a genuine endogenous neurobiological mechanism and to characterize the potential underlying pathways.

## Plain Language Summary

Vomiting during a migraine is often seen as a debilitating symptom, but in our questionnaire‐based study, we found that more than half of people with migraine who vomit during an attack experience meaningful pain relief afterward, and for some, pain disappears within minutes. Some people reported intentionally inducing vomiting as a way to manage their migraine attacks. Better understanding why and how vomiting can cut a migraine attack short could offer important clues about the underlying mechanisms of migraine.

**Abbreviations**
CeA: central nucleus of the amygdala
CGRP: calcitonin gene‐related peptide
DVC: dorsal vagal complex
ICHD‐3: International Classification of Headache Disorders, 3rd edition
IQR: interquartile range
lPBN: lateral parabrachial nucleus
NTS: nucleus tractus solitarius
VIMPR: vomiting‐induced migraine pain relief

## INTRODUCTION

Migraine is a neurological disorder representing a leading cause of disability, particularly among individuals under 50 years of age.^1^, ^2^ Even though cephalic pain is the most debilitating symptom during the migraine attack, the accompanying features such as nausea, vomiting, photophobia, and phonophobia often are equally debilitating for patients (namely bothersome symptoms).^3^ Specifically, nausea occurs in approximately 90% of migraine attacks, whereas vomiting affects 30%–70% of patients,^4^ making these symptoms integral to the International Classification of Headache Disorders, 3rd edition (ICHD3) diagnostic criteria,^5^ and responsible for the increase of the clinical burden.^6^ The high prevalence of nausea and vomiting during migraine attacks, along with the occurrence of nausea before pain, suggests an intricate pathophysiologies underlying the genesis of these symptoms that still needs to be clarified. In particular, the presence of nausea before the headache phase suggests that it could be triggered by primary brain dysfunction rather than being secondary to trigeminovascular nociceptive input.^7^

In clinical practice, symptomatic medications are routinely used to counteract nausea and vomiting in patients with migraine. However, it is often overlooked that patients may experience significant pain relief on vomiting (thereafter defined as vomiting‐induced migraine pain relief [VIMPR]).^8^ This phenomenon, although anecdotally recognized by clinicians and patients for centuries (Hippocrates first reported the occurrence of VIMPR),^9^ has neither been carefully described at the clinical level, nor investigated in terms of underlying neurochemistry and circuit‐based mechanisms. Indeed, although some hypotheses have been proposed,^8^ there is lack of information on the neurobiology of VIMPR.

Evidence of VIMPR derives from two observational studies reporting vomiting as a pain‐relieving event in patients with migraine.^10^, ^11^ The analgesic effects of vomiting during a migraine attack have also been described in a study investigating the pathophysiological role of cranial autonomic symptoms in migraine.^12^

However, these studies^10^, ^11^ relied on qualitative assessments within the context of broader investigations into behaviors used to relieve pain during migraine attacks, without providing quantitative data regarding changes in pain intensity, as well as the temporal kinetics of the onset and duration of VIMPR.

Based on the limited prior literature available and the clinical observation that migraine attacks accompanied by vomiting are typically more severe, we hypothesized that: (1) VIMPR would occur in a considerable proportion of patients experiencing emesis during attacks; (2) the temporal onset of pain relief would demonstrate patterns inconsistent with spontaneous attack resolution; and (3) the behavior of voluntary vomiting induction would be associated with patients' prior experience of VIMPR.

Here, we report an exploratory, questionnaire‐based study conducted in patients with migraine with the aim to characterize the prevalence, temporal patterns, and extent of VIMPR, as well as to investigate behaviors related to voluntary vomiting induction as a potential self‐treatment strategy.

## METHODS

### Study design and setting

This cross‐sectional questionnaire‐based study was conducted from June 2023 to December 2023 at the Headache Center of Careggi University Hospital, Florence. The study was reported in accordance with the Strengthening the Reporting of Observational Studies in Epidemiology (STROBE) guidelines.^13^ The study represents a secondary, not–preplanned analysis of data collected within the Registro Italiano Cefalee (RICe) study, which was approved by the Comitato Etico Area Vasta Centro (CEAVC Studio RICe, 14591_oss and subsequent amendments). Written informed consent was obtained from all participants before questionnaire administration and covered under the general registry approval.

### Participants

Inclusion criteria were ≥18 years old, informed consent to participate, and fulfillment of the criteria of diagnosis of migraine without aura, migraine with aura, or chronic migraine (CM) according to ICHD‐3.^5^ Patients with medication‐overuse headache (MOH), according to ICHD‐3 criteria, were included as the questionnaire focused on vomiting characteristics during migraine attacks regardless of medication‐overuse status. Patients were excluded if they had any combination of migraine with other primary headache disorders, as well as those with secondary headache disorders (excluding MOH).

### Variables and data collection

During the study period, all consecutive patients attending the outpatient clinic who met inclusion criteria were approached and invited to participate (*n* = 125). Of these, seven patients declined participation and 12 provided incomplete responses, resulting in 106 patients included in the final analysis. Patients were screened sequentially during routine clinical visits without pre‐selection. The questionnaire was administered during the clinic visit by trained personnel who explained the study purpose and obtained written informed consent before data collection. All participants included in the study completed an *ad hoc* structured questionnaire (see Supporting Information), specifically developed to assess the relationship between vomiting and pain characteristics during migraine attacks.

The questionnaire followed a structured sequence administered by trained personnel. The initial screening question asked whether the patient experienced vomiting during migraine attacks. For patients responding negatively, no further vomiting‐related questions were administered. For patients reporting vomiting episodes, the interviewer assessed vomiting frequency during monthly attacks using a categorical scale, number of vomiting episodes per single attack, whether attacks with vomiting were more painful than usual, and pain intensity using numerical rating scales ranging from 0 (no pain) to 10 (worst imaginable pain) for attacks with and without vomiting. To ensure consistent interpretation, trained personnel provided patients with standardized numerical ranges for each frequency category during questionnaire administration: “rarely” was defined as fewer than one attack with vomiting per month (<1/month), “sometimes” as one to three attacks per month, “often” as four to eight attacks per month, “very often” as more than eight attacks per month (>8/month), and “always” as vomiting occurring in every migraine attack. Additional assessments included temporal sequence of vomiting relative to pain onset, pain reduction or cessation following vomiting episodes (namely VIMPR), timing of pain relief (seconds, minutes, or hours), pain recurrence patterns, and voluntary vomiting induction behaviors as a pain management strategy. Demographic and clinical characteristics (i.e., age, sex, migraine type, presence of aura, medication‐overuse) were collected. Information on acute medication use (including triptans, NSAIDs, and antiemetics) during individual migraine attacks was not available.

### Statistical analysis

Because of the exploratory and descriptive nature of the study, no formal sample size calculation was performed. A convenience sample was used, with the sample determined by all patients meeting inclusion criteria presenting to the Headache Center during the study period. The analysis includes all the patients who completed the questionnaire (*n* = 106). All patients who reported experiencing vomiting during attacks provided complete responses to questions about vomiting characteristics, pain intensity comparisons, and VIMPR. Complete case analysis was used throughout.

Categorical variables were presented as absolute frequencies and corresponding percentages, reported as *n*/*N* (percentage). For key prevalence estimates, 95% confidence intervals (CIs) were calculated using the Wilson score method. Continuous variables were summarized descriptively, using either mean ± standard deviation or median and interquartile ranges (IQR) as appropriate. Normality of continuous variables was assessed using the Shapiro–Wilk test. Because of a non‐normal distribution and the paired nature of the data, the Wilcoxon signed‐rank test for paired data was used for comparing pain intensity between attacks with and without vomiting within the same patients. For comparisons of pain intensity across multiple groups (number of vomiting episodes per attack), the Kruskal–Wallis test was used. For age comparison between patients with and without vomiting, the independent samples Welch's *t*‐test was used. Associations between categorical variables were examined using Pearson's χ^2^ test with Yates' continuity correction for 2 × 2 tables; Fisher exact test was applied when any expected cell count was <5.

Degrees of freedom are reported for all χ^2^ and Kruskal–Wallis tests. All statistical tests were two‐tailed with statistical significance set at *p* < 0.05. Data management and statistical analyses were performed using R version 4.4.0 (R Foundation for Statistical Computing, Vienna, Austria) using the stats package (base R) for Shapiro–Wilk, Wilcoxon signed‐rank, Kruskal–Wallis, χ^2^ (with Yates' correction for 2 × 2 tables), and Fisher's exact tests, and the *DescTools* package for Wilson score confidence intervals for single proportions.

## RESULTS

### Participant characteristics and vomiting prevalence

**FIGURE 1:**
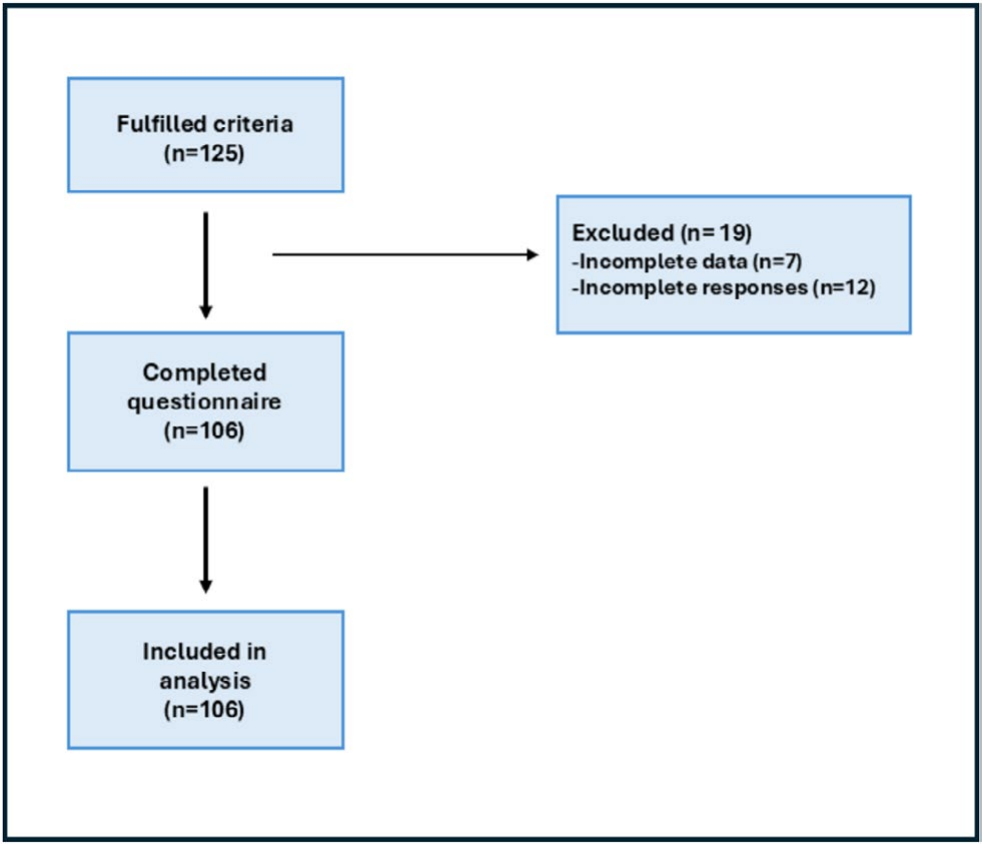
Flowchart of patients. A total of 125 patients fulfilled inclusion criteria during the study period. Of 19 patients who did not complete the questionnaire, seven declined participation and 12 provided incomplete responses. The final analysis included 106 patients who provided complete questionnaire data. [Color figure can be viewed at wileyonlinelibrary.com]

A total of 125 patients fulfilled the inclusion criteria during the study period. Of these, 106 patients completed the questionnaire and were included in the analysis. Reasons for exclusion and the number of patients included are reported in Figure 1. The study population consisted predominantly of females (91 patients, 85.8%) with a mean age of 43.4 ± 13.6 years (range, 18–75 years) including 87 of 106 patients with episodic migraine (82.1%), 19 of 106 with CM (17.9%), and among them, 20 of 106 with migraine with aura (18.9%). In the patients with CM, 14 of 19 (73.6%) had MOH. Demographic and baseline headache characteristics of patients included are reported in Table 1. Eighty‐two patients (82 of 106, 77.4%; 95% CI, 68.5%–84.3%) reported experiencing vomiting during migraine attacks. Vomiting prevalence did not differ significantly between patients with episodic migraine and CM (70 of 87, 80.5% vs. 12 of 19, 63.2%; Fisher exact test, *p* = 0.131). Demographic and clinical characteristics were comparable between patients with and without vomiting (Table 2).

**TABLE 1:** Demographic and clinical characteristics of study participants (*n* = 106).

	Patients (*n* = 106)
Demographics	
Age, years (mean ± SD)	43.4 ± 13.6
Female sex, *n* (%)	91 (85.8)
Migraine features	
Episodic migraine, *n* (%)	87 (82.1)
Chronic migraine, *n* (%)	19 (17.9)
Aura, *n* (%)	20 (18.9)
Medication‐overuse headache, *n* (%)	14 (13.2)

*Note*: Data are presented as mean ± SD for continuous variables and as *n* (%) for categorical variables. SD, standard deviation.

**TABLE 2:** Comparison of demographic and clinical characteristics between patients with and without vomiting during migraine attacks.

	Vomiting (*n* = 82)	No vomiting (*n* = 24)	*p* value
Demographics			
Age, years (mean ± SD)	42.8 ± 13.4	45.5 ± 14.2	0.412*
Female sex, *n* (%)	71 (86.6)	20 (83.3)	0.741
Migraine features			
Episodic migraine, *n* (%)	70 (85.4)	17 (70.8)	0.131
Chronic migraine, *n* (%)	12 (14.6)	7 (29.2)	
Aura, *n* (%)	16 (19.5)	4 (16.7)	1.000
Medication‐overuse headache, *n* (%)	10 (12.2)	4 (16.7)	0.516

*Note*: Patients were stratified according to self‐reported occurrence of vomiting during migraine attacks. No statistically significant differences were observed between groups for any demographic or clinical characteristic. SD, standard deviation.

* Independent samples *t*‐test (Welch's correction); Fisher exact test for categorical comparison due to expected cell counts <5. Data are presented as mean ± SD for continuous variables and as *n* (%) for categorical variables.

### Characteristics of vomiting during migraine attacks

**FIGURE 2:**
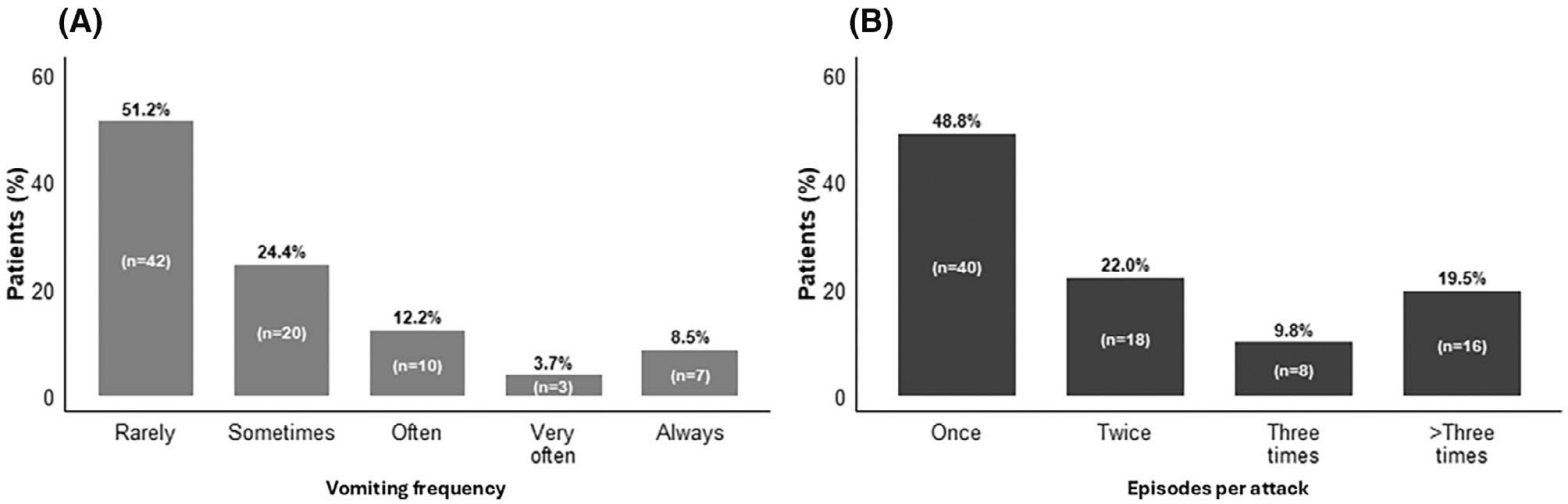
Vomiting characteristics in patients with migraine. (A) Self‐reported frequency of vomiting in monthly migraine attacks. The majority of patients (51.2%) reported vomiting rarely, whereas 8.5% reported vomiting during every attack. (B) Number of vomiting episodes per migraine attack. Nearly half of patients (48.8%) experienced a single episode, whereas 19.5% reported more than three episodes per attack. Data are presented as percentages with absolute counts (*n*) shown within bars.

Among patients who experienced vomiting (*n* = 82), the frequency pattern during monthly attacks was as follows: rarely in 42 patients (51.2%), sometimes in 20 patients (24.4%), often in 10 patients (12.2%), very often in three patients (3.7%), and always in seven patients (8.5%) (Figure 2A).

Regarding the number of vomiting episodes during the attack, patients (*n* = 82) reported a single (40 patients, 48.8%), two (18 patients, 22.0%), three (eight patients, 9.8%), and more than three (16 patients, 19.5%) episodes (Figure 2B).

### Comparison of pain intensity between attacks with and without vomiting

**FIGURE 3:**
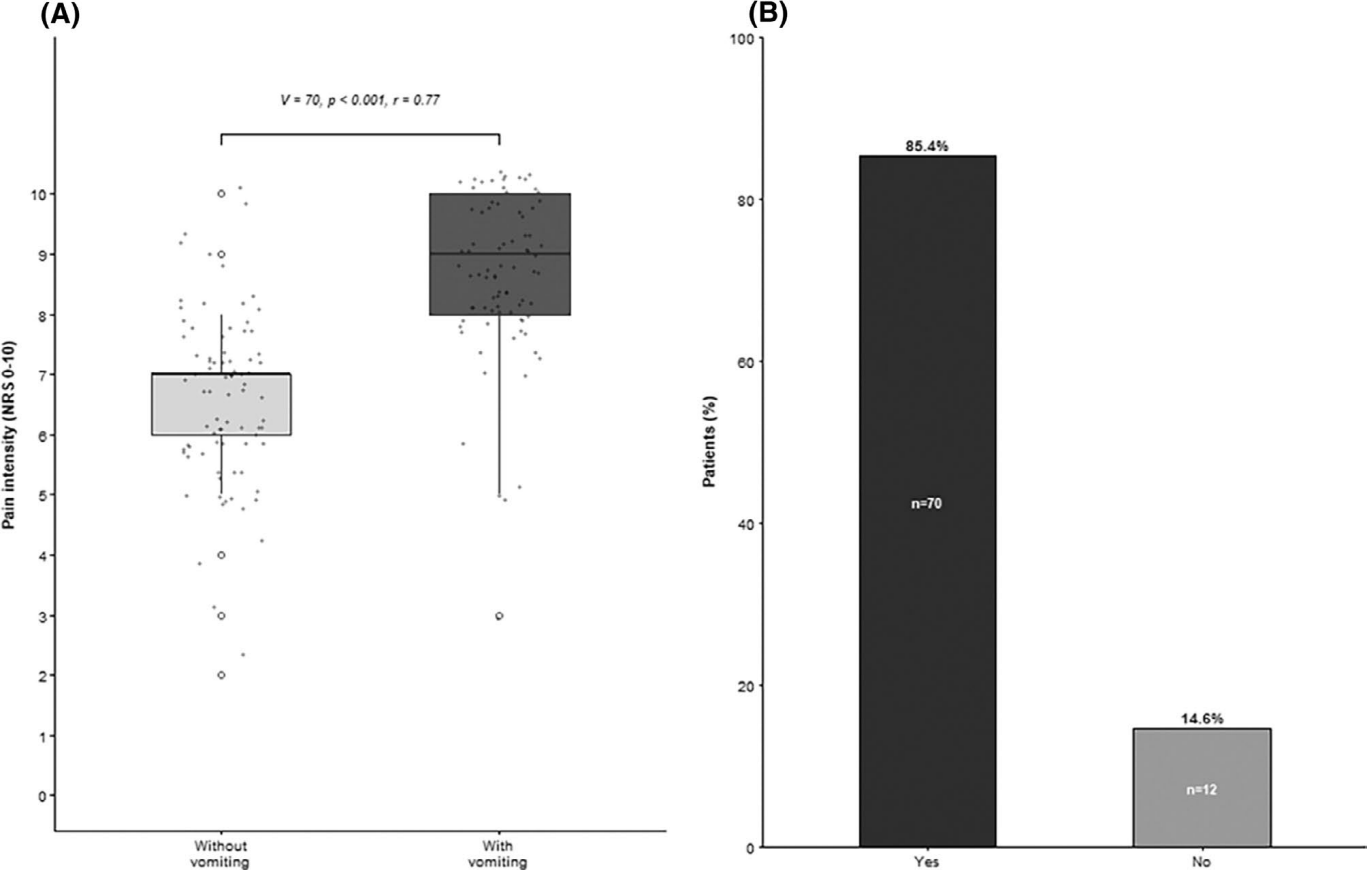
Pain characteristics in migraine attacks with and without vomiting. (A) Comparison of self‐reported pain intensity between migraine attacks without vomiting and attacks accompanied by vomiting within the same patients. Pain was assessed using the NRS, 0–10. Box plots showing pain intensity distribution on NRS scale for attacks without vomiting (median, 7.0; IQR, 6.0–7.0) versus attacks with vomiting (median, 9.0; IQR, 8.0–10.0). Statistical comparison performed using Wilcoxon signed‐rank test for paired data: *V* = 70, *p* < 0.001, *r* = 0.77. (B) Perceived pain severity. Patients were asked whether migraine attacks accompanied by vomiting were perceived as more painful than attacks without vomiting. The majority (85.4%) confirmed that vomiting‐associated attacks were more painful. IQR, interquartile range; NRS, numerical rating scale; effect size (*r*) calculated as Z/N.

Median pain intensity on the numerical rating scale was 7.0 (IQR, 6.0–7.0) and 9.0 (IQR, 8.0–10.0) for attacks without and with vomiting, respectively. Wilcoxon signed‐rank test for paired data showed a statistically significant difference in pain intensity (*V* = 70.00, *p* < 0.001, effect size *r* = 0.77) (Figure 3A). This quantitative assessment is in line with the patients' perception: 70 of 82 patients (85.4%) reported that attacks with vomiting were more painful than those without emesis, whereas only 12 of 82 patients (14.6%) perceived no difference in pain intensity (Figure 3B). Among patients experiencing vomiting, pain intensity did not significantly differ by number of episodes per attack (Kruskal–Wallis test: *H* (3) = 5.86, *p* = 0.119).

### Pain relief following vomiting

**FIGURE 4:**
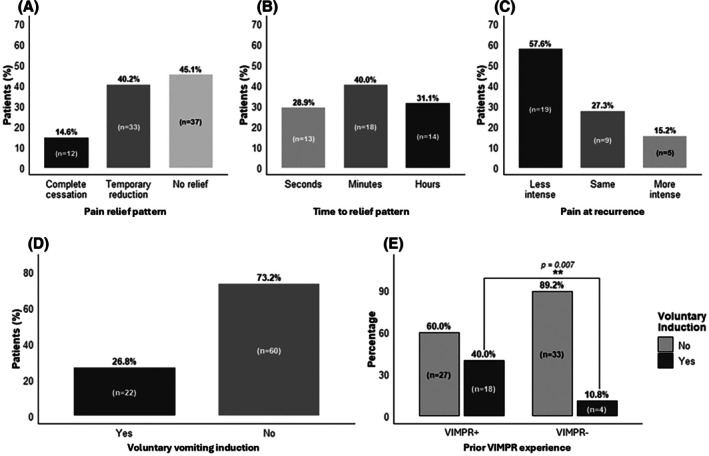
Clinical features of VIMPR and voluntary vomiting induction behavior. (A) Pain relief pattern following vomiting among all patients who reported vomiting during attacks (*n* = 82). Complete cessation indicates total resolution of headache following vomiting without subsequent recurrence; temporary reduction indicates partial relief with subsequent pain recurrence; no relief indicates absence of any perceived analgesic effect. (B) Temporal pattern of VIMPR onset among patients experiencing pain relief (*n* = 45). Time categories represent patient‐reported intervals between vomiting and onset of pain relief: seconds, minutes, or hours. (C) Pain intensity at recurrence compared to pre‐vomiting levels among patients experiencing temporary reduction with subsequent pain recurrence (*n* = 33). The majority (57.6%) reported reduced intensity upon recurrence compared to pre‐vomiting pain levels. (D) Prevalence of voluntary vomiting induction as a self‐treatment strategy among patients who reported vomiting during attacks (*n* = 82). Over one‐quarter (26.8%) reported deliberately inducing vomiting to obtain pain relief. (E) Association between prior VIMPR experience and voluntary vomiting induction behavior. Patients with prior VIMPR experience (VIMPR+) were significantly more likely to report voluntary induction compared to those without prior relief (VIMPR–): 40.0% versus 10.8% (χ^2^(1) = 7.39, *p* = 0.007). This association suggests that voluntary induction represents a learned self‐treatment behavior based on prior therapeutic experience. IQR, interquartile range; VIMPR, vomiting‐induced migraine pain relief; VIMPR+, patients with prior VIMPR experience; VIMPR−, patients without prior VIMPR experience.

Regarding the analgesic effect of vomiting, 45 of 82 patients (54.9%; 95% CI, 44.1%–65.2%) reported benefit after emesis. Specifically, among these 45 patients, 12 of 45 (26.7%; 95% CI, 16.0%–41.0%) reported pain freedom, whereas 33 of 45 (73.3%; 95% CI, 59.0%–84.0%) reported only temporary pain relief with subsequent recurrence (Figure 4A). Notably, we also evaluated the temporal pattern of VIMPR onset, finding that 13 of 45 patients (28.9%; 95% CI, 17.7%–43.4%) reported pain relief within seconds, 18 of 45 (40.0%; 95% CI, 27.0%–54.5%) within minutes (median, 15.0; IQR, 6.2–27.5 min), and 14 of 45 patients (31.1%; 95% CI, 19.5%–45.7%) within hours (median, 2.0; IQR, 2.0–2.8 h) (Figure 4B).

We also asked patients to compare pain intensity after VIMPR to that before vomiting. Among the 33 patients experiencing pain recurrence, 19 of 33 (57.6%; 95% CI, 40.8%–72.8%) reported reduced pain intensity, nine of 33 (27.3%; 95% CI, 15.1%–44.2%) reported unchanged intensity, and five of 33 (15.2%; 95% CI, 6.7–30.9%) reported increased intensity (Figure 4C). In exploratory analysis, we examined whether the timing of initial pain relief influenced recurrence patterns, hypothesizing that relief occurring after hours might represent natural attack resolution rather than true VIMPR. When restricting analysis to the 24 patients with rapid‐onset relief (seconds to minutes), who subsequently experienced pain recurrence, no statistically significant difference was observed between patients with second‐onset versus minute‐onset relief regarding favorable pain recurrence patterns defined as reduced pain intensity (4 of 11, 36.4% vs. 9 of 13, 69.2%; Fisher exact test, *p* = 0.217), although the analysis was limited by the small sample size.

### Voluntary vomiting induction

Considering the analgesic effects of VIMPR, 22 of 82 patients (26.8%; 95% CI, 18.4%–37.3%) who experienced vomiting reported intentional induction of vomiting (Figure 4D). This behavior appeared to be influenced by previous experience of the analgesic benefit. Indeed, voluntary induction of emesis was reported by 18 of 45 patients (40.0%; 95% CI, 27.0%–54.5%) who had experienced VIMPR and four of 37 (10.8%; 95% CI, 4.3–24.7) of those reporting lack of VIMPR (χ^2^(1) = 7.39, *p* = 0.007) (Figure 4E).

## DISCUSSION

The present study revealed that VIMPR is reported by 54.9% of patients with migraine, ranging from temporary pain relief to pain freedom. Our data show clinical duality of vomiting: it usually correlates with the more severe attacks but could provide pain relief.

Our study builds on prior work reporting spontaneous or induced vomiting as a pain‐relieving event in migraine but not in patients with tension‐type headache, according with diagnosis criteria.^10^, ^11^ Data are also consistent with another study showing that emesis triggers analgesia in patients with migraine regardless of the presence or absence of cranial autonomic symptoms.^12^ For the first time, we provide a detailed description of the incidence, temporal patterns, duration and analgesic efficacy of VIMPR. Notably, a relationship between pain and vomiting is well established in the clinical setting, but it typically relates to the ability of pain to trigger emesis in particular conditions such as severe, acute somatic, or visceral pain. To our knowledge, the opposite relationship, namely the analgesic effect of emesis, has not been reported for pain disorders other than migraine. Notably, vomiting represents a characteristic symptom integral to migraine diagnostic criteria, rather than only a consequence of pain severity, which may explain why this relationship has been observed specifically in this condition.

The neurophysiological mechanisms underlying VIMPR remain unknown.^8^ Over a quarter of patients in our study auto‐induce vomiting as an analgesic strategy, and we found that this procedure is more common among those with prior experience of VIMPR, thereby suggesting a form of empirically driven self‐treatment practice. Although this association is expected from a behavioral perspective, it provides indirect support for a genuine therapeutic effect of VIMPR, suggesting that the reported pain relief reflects a reproducible phenomenon rather than coincidental attack resolution in selected patients. Moreover, the reported rapid onset of VIMPR, if confirmed by prospective studies, would support the involvement of active, neurophysiological mechanisms. Indeed, although delayed pain relief (hours) might reflect coincidental attack resolution, a substantial proportion of patients reported VIMPR onset within seconds or minutes, timeframes that would be inconsistent with spontaneous recovery. Although these self‐reported temporal patterns should be interpreted cautiously given the inherent limitations of retrospective recall and the use of analgesics, this effects could be related to the activation of antinociceptive projection pathways originating from structures of the dorsal vagal complex (DVC) such as the area postrema, the nucleus tractus solitarius (NTS), and the dorsal motor nucleus of the vagus.^14^ Autoradiographic and retrograde tracer experiments show that the NTS projects to limbic and hypothalamic structures, such as the central nucleus of the amygdala as well as the paraventricular and dorsomedial hypothalamic nuclei, key centers for both autonomic regulation and nociceptive modulation.^15^ More recently, an exploratory positron emission tomography study^7^ provided direct neuroimaging evidence of the activation of these interconnected critical nuclei specifically in patients who experienced nausea during the premonitory phase of nitroglycerin‐induced migraine compared to those without nausea. The results demonstrate significant activation of central structures in the rostral dorsal medulla, including NTS, dorsal motor nucleus of the vagus, and nucleus ambiguous, along with the periaqueductal gray (PAG) in patients experiencing nausea. Notably, these activations occurred in the absence of pain during the premonitory phase.^7^ This raises the critical question of why nausea, which is nearly ubiquitous in migraine attacks and highly bothersome,^6^ does not provide the same analgesic benefit as vomiting, despite activating similar brainstem structures. To answer this question, we propose the “magnitude hypothesis” claiming that whereas the upper dorsal medulla and PAG are activated both during nausea and vomiting,^7^ the vomiting reflex occurs when a much more robust and coordinated activation of these same structures takes place. Indeed, the act of vomiting involves synchronized motor outputs engaging multiple cranial nerves, vagal efferent discharge, and autonomic changes that might activate descending, antinociceptive circuits.^16^ Future studies employing imaging techniques to compare brain activation patterns before and after vomiting episodes versus patients experiencing nausea alone could provide indirect evidence for this hypothesis.

An additional projection pathway that might be involved in VIMPR is operated by calcitonin gene‐related peptide (CGRP) neurons of the parabrachial nucleus.^17^ Preclinical studies demonstrate that the lateral parabrachial nucleus (lPBN), a key hub of pain processing, receives strong input from the NTS and sends glutamatergic projections to the central nucleus of the amygdala (CeA), another key center of nociception.^18^ The amygdala not only regulates the emotional response to pain, but also pain intensity via emotional regulation, two events strictly interconnected with the genesis of migraine. Notably, the lPBN‐to‐CeA projections co‐release CGRP and pituitary adenylate cyclase‐activating polypeptide,^19^ sustaining nociplastic pain, including cephalic allodynia induced by nitroglycerin injection in mice,^18^ a prototypical experimental model of migraine pain. Further emphasizing a possible involvement of the parabrachio–amygdala pathway in migraine pain and VIMPR, recent evidence in rats indicates that nausea sustained by lPBN neurons releasing CGRP in the amygdala can be counteracted by peripheral administration of anti‐CGRP monoclonal antibodies used for migraine prophylaxis such as fremanezumab or galcanezumab.^20^ To note, nausea is the symptom that is more rapidly reduced (within 30 min) in patients with migraine receiving the anti‐CGRP mAb eptinezumab intravenously.^21^ Therefore, the lPBN‐to‐CeA projections could transmit both nausea and pain signals during migraine, and output from the DVC during emesis that typically relieves nausea might also counteract the nociceptive component, thereby leading to pain relief.

The functional connection between the DVC and the migraine pain pathway might be a neuroanatomical peculiarity present in some patients with migraine.^22^ Whether this represents a common evolutionary feature or a migraine‐specific phenomenon needs to be evaluated, considering that vomiting‐induced analgesia has not yet been documented in other pain disorders associated with emesis, and that individuals experiencing vomiting triggered by nonpainful stimuli (e.g., toxic ingestions) would have no baseline pain to relieve.

Additionally, or alternatively, in light of the rapid onset of analgesia after emesis, activation of the descending noxious inhibitory control system might underlie VIMPR. Key hubs of the pain matrix participating to descending noxious inhibitory control such as the nucleus raphe magnus and the PAG are activated during vomiting.^23^ Even the activation of the insular and somatosensitive cortices during vomiting might trigger top‐down feedback mechanisms known to counteract meningeal nociception within the trigeminal nucleus.^24^ Finally, the massive parasympathetic activation that typically characterizes the vomiting reflex might concur to VIMPR. Indeed, the parasympathetic output occurring during emesis might functionally reproduce the noninvasive vagus nerve stimulation adopted in patients with migraine to prompt pain relief.^25^, ^26^

Research on migraine has primarily focused on endogenous triggers rather than suppressors of pain.^27^ Although sleep represents the most recognized endogenous suppressor, it is worth noting that it works through gradual attack termination, a process that unfolds over hours,^28^, ^29^ whereas VIMPR appears to operate immediately, providing therapeutic benefit within seconds to minutes. To our knowledge, the only procedure able to suppress migraine pain within seconds or minutes is subcutaneous sumatriptan.^30^ Although the effect of vomiting on brainstem 5HT1_D/F_ receptor expressing neurons is unknown, some evidence indicates that triptans could reach the brain parenchyma.^31^, ^32^, ^33^ Whether VIMPR and triptans share neurochemical mechanisms involved in migraine pain suppression remains a speculative hypothesis that warrants further investigation, considering that nausea is also an adverse event related to triptans.^34^

VIMPR might also be ascribed to meningeal vasoactive mechanisms. Nausea is indeed typically associated with cutaneous vasoconstriction, whereas vomiting is associated with stereotypic autonomic responses where sympathetic hyperactivity with increased heart rate and peripheral vasoconstriction occurs.^35^ Thus, it will be worth investigating whether meningeal vessels of patients with migraine undergo constriction upon vomiting or, conversely, dilate because of the concomitant parasympathetic activation typically causing sialorrhea and lacrimation.

An important question for future investigation concerns whether individuals with childhood‐onset migraine, characterized by prominent gastrointestinal symptoms,^36^ continue to experience vomiting in adulthood and, if so, whether they are more likely to experience VIMPR. Longitudinal studies following pediatric migraine cohorts into adulthood could determine whether early vomiting patterns predict adult VIMPR susceptibility, potentially identifying a stable neurobiological phenotype.

Some limitations warrant consideration in interpreting our findings. First, as an exploratory investigation relying on an *ad hoc*, nonvalidated questionnaire, this research is based exclusively on retrospective self‐reports, introducing potential for recall bias, as participants may preferentially remember episodes where vomiting was followed by pain relief. Moreover, patients may have difficulty precisely recalling the timing of pain relief (distinction between relief occurring within seconds vs. minutes) during episodes characterized by severe pain. An additional limitation concerns the lack of data on acute medication use during individual migraine attacks. Indeed, triptans may induce vomiting as an adverse effect while simultaneously providing pain relief within the same timeframe observed for VIMPR, introducing a potential confound whereby patients could attribute improvement to vomiting rather than to medication. Without attack‐level information on medication timing and dosing, it is not possible to disentangle drug‐related relief from endogenous VIMPR or to determine whether medication use modulates the phenomenon. Fourth, recruitment from a single headache center introduces selection bias and limits the generalizability of our findings, particularly with respect to the observed vomiting prevalence, which exceeds rates reported in population‐based studies.^4^ The relatively small sample size, although adequate for descriptive analyses, further limits detailed subgroup characterization. Finally, the absence of objective neurobiological measures (e.g., neuroimaging and monitoring of neurophysiological parameters) precludes detailed, mechanistic interpretations.

## CONCLUSIONS

In this exploratory questionnaire‐based study, we found that vomiting is associated with pain relief or pain freedom in over half of patients with migraine who experience emesis during attacks. The rapid onset of VIMPR reported by patients and the learned behavior of voluntary vomiting induction are consistent with the involvement of endogenous antinociceptive mechanisms. However, the cross‐sectional and self‐reported nature of our data does not allow us to establish whether this phenomenon reflects the activation of neurobiological pathways, spontaneous attack resolution, delayed effects of acute medications, or a combination of these factors. To better understand the role of vomiting in migraine pathophysiology, future studies should prioritize multimodal approaches combining prospective data collection, ideally complemented by neurophysiological/neuroimaging monitoring.

## AUTHOR CONTRIBUTIONS

**Antonio Munafò:** Investigation; formal analysis; writing – original draft; writing – review and editing. **Andrea Burgalassi:** Investigation; visualization. **Giulia Vigani:** Investigation; visualization. **Luigi Francesco Iannone:** Investigation; methodology; writing – review and editing; visualization; data curation. **Francesco De Cesaris:** Writing – review and editing. **Alberto Chiarugi:** Conceptualization; writing – original draft; writing – review and editing; methodology; supervision.

## FUNDING INFORMATION

This research did not receive any specific grant from funding agencies in the public, commercial, or not‐for‐profit sectors.

## CONFLICT OF INTEREST STATEMENT

**Luigi Francesco Iannone** received financial support, consulting fees for the participation in advisory boards and support for attending meetings from: Teva, Eli Lilly, Lundbeck, Pfizer and AbbVie; he is Associate Editor for Frontiers and junior editor of Cephalalgia and Cephalalgia report. **Francesco De Cesaris** received personal fees from TEVA, Eli Lilly, Novartis, and AbbVie. **Alberto Chiarugi** received personal fees from TEVA and Eli Lilly. **Antonio Munafò, Andrea Burgalassi**, and **Giulia Vigani** have no relevant financial or nonfinancial interests to disclose.

## Supporting information


Data S1:

## Data Availability

Data are available from the corresponding author on reasonable request.
