# Supplementary material for: Beyond symptom: Exploring the analgesic properties of vomiting in patients with migraine
Source: Headache. 2026 May 27;66(6):1376–85. doi: 10.1111/head.70106 (PMC13282612; doi:10.1111/head.70106)
Supplement: Supplementary file 1 — Data S1: [file HEAD-66-1376-s001.pdf]

QUESTIONNAIRE

Patient Demographics

- Age: _______
- Gender: □ Female □ Male

1. **Do you sometimes vomit during a migraine attack?** □ Yes □ No

[If NO, the questionnaire ends here]

2. Of all the migraine attacks you have per month, how often does vomiting occur?

□ Rarely □ Sometimes □ Often □ Very often □ Always

3. Are migraine attacks that occur with vomiting more painful than usual attacks? □ Yes □ No

4. Pain intensity of migraine attacks without vomiting (0-10 scale): _______

5. Pain intensity of migraine attacks with vomiting (0-10 scale): _______

6. How many times do you vomit during a single migraine attack?

□ Once □ Twice □ Three times □ More than three times

7. When you vomit, has the pain (headache) already started? □ Yes □ No

8. After vomiting, does the pain reduce/stop? □ Yes □ No

9. If the pain reduces or stops, how long does it take for the pain to reduce/stop?

□ Seconds □ Minutes → How many minutes? _______ □ Hours → How many hours? _______

10. Does it happen that after the pain reduction/cessation, the pain starts again? □ Yes □ No

11. After how long does the migraine pain start again?

□ Seconds □ Minutes → How many minutes? _______ □ Hours → How many hours? _______

12. If the migraine pain starts again, how is the pain compared to before vomiting?

□ Less intense □ Same □ More intense

13. Intensity of migraine pain when it starts again after vomiting (0-10 scale): _______

14. Do you ever try to induce vomiting during an attack to try to reduce the migraine pain?
□ Yes □ No
